# Factors Associated With Emergency Department Visits or Readmission of Late Preterm Infants at the Neonatal Intensive Care Department, National Guard Health Affairs, Riyadh

**DOI:** 10.7759/cureus.37604

**Published:** 2023-04-15

**Authors:** Abdurhman S Alsaif, Khalid A Almutairi, Nawaf D Aljehani, Eid D Alanazi, Abdullah Alqahtani, Aly F Mahmoud

**Affiliations:** 1 College of Medicine, King Saud Bin Abdulaziz University for Health Sciences, Riyadh, SAU; 2 Neonatal Intensive Care Department, King Abdulaziz Medical City, Ministry of National Guard Health Affairs, Riyadh, SAU

**Keywords:** git problems, urti, sepsis, jaundice, late preterm infants

## Abstract

Background

Infants who are born between 34 0/7 and 36 6/7 weeks of pregnancy as a result of maternal or fetal factors are defined as “late preterm infants”. Compared to term infants, late preterm infants are more predisposed to pregnancy complications because they are less mature physiologically and metabolically. In addition, health practitioners still face difficulties in differentiating between term and late preterm infants due to similar general appearance. The aim of this study is to explore the epidemiology of readmission among late preterm infants at the National Guard Health Affairs. The objectives of the study were to calculate the rate of readmission among late preterm infants in the first month after discharge and to identify the associated risk factors for readmission.

Methods

A retrospective cross-sectional study was carried out at the neonatal intensive care unit (NICU at King Abdulaziz Medical City in Riyadh). We identified preterm infants born in 2018 and the risk factors for readmission within the first month of life. Data on risk factors were collected using the electronic medical file.

Results

A total of 249 late preterm infants with a mean gestational age of 36 weeks were included in the study. Of them, 64 infants (25.7%) suffered from at least a subsequent admission and stayed overnight in either the inpatient department or pediatric emergency room. Maternal diabetes was a significant risk factor for readmission; on the other hand, a positive maternal Rh factor was a protective factor against readmission. Among readmitted infants (n=64), 51 infants were admitted to the emergency room (79.69%), eight infants were readmitted to the pediatric ward (12.5%), and five infants were readmitted to both (7.8%). The most common cause for pediatric ER visits was gastrointestinal (GIT) problems (27%), followed by upper respiratory tract infection (URTI) (18%) and jaundice (14%). The most common cause for direct ward readmission was jaundice (n= 5; 62%).

Conclusion

Gastrointestinal (GIT) issues and upper respiratory tract infections (URTIs) were the leading causes of pediatric emergency room admissions. In contrast, jaundice, congenital diaphragmatic hernia (CDH), airway problems, and regurgitation were the most frequent causes of admission to the ward, with jaundice being the primary cause. Although studies suggest that the late preterm population is at a higher risk for long-term health issues, further research is necessary to investigate this topic thoroughly.

## Introduction

Infants who are born between 34 0/7 and 36 6/7 weeks of pregnancy as a result of maternal or fetal factors are defined as “late preterm infants” [1]. Compared to term infants, late preterm infants are more predisposed to pregnancy complications because they are less mature physiologically and metabolically. In addition, health practitioners still face difficulties in differentiating between term and late preterm infants due to similar general appearance. In the United Kingdom, late preterm infants represent approximately three-quarters of all premature births; yet they have been studied much less than their more immature counterparts born at the limits of viability [2].

Lately, studies concerning late preterm infants (LPIs) have been rising significantly in the Kingdom of Saudi Arabia (KSA). However, local research are still insufficient in terms of quantity when compared to international studies. As per a study conducted at the Department of Pediatrics at King Abdulaziz University, late preterm infants’ mothers were found to have older age, higher parity, BMI, and increased number of multiple pregnancies which increase the incidence of late preterm delivery. Nevertheless, they did not vary from mothers of term infants in educational background, working status, and smoking [1]. Although a recent systematic review found that most late preterm infants (LPIs) show positive physical and developmental outcomes in the short and long term, their sheer volume results in a substantial burden on healthcare costs [3].

The rate of readmission of preterm infants is associated with several factors such as jaundice, feeding problems, respiratory distress, and sepsis evaluation. According to previous studies that were performed in the past, newly born late preterm infants are usually nurtured and taken care of before getting discharged from the hospital when they reach the postnatal age of two to three days [4]. Also, it was illustrated in past studies that the likelihood for late preterm infants to be readmitted is three times more than for term infants for the previous risk factors [5]. Moreover, a study indicated that 70% of causes of readmission of late preterm infants are respiratory diseases with a median interquartile range (IQR) of hospital stay was 7 (6-10) days [6].

Late preterm infants are medically cleared earlier than other corresponding preterm neonates because they meet the criteria for discharge. Many studies indicate that the relationship between the length of stay (LOS) and readmission is inversely proportional [7]. Therefore, the risk of readmission for late preterm babies decreases by 8.6% for every additional day of stay. According to some studies, in cesarean births, no connection was discovered between LOS and readmission. In contrast to vaginal birth, each additional day of newborn stay was associated with a 3.0% increase in the adjusted risk of readmission [8]. It was illustrated that due to certain respiratory illnesses such as wheezing or bronchiolitis during the first two years of life, late preterm infants in comparison with full-term infants are at high risk of readmission. Nevertheless, there was no significant difference in the severity of the illness during hospitalization and readmission [9].

In the last decade, the rate of preterm infants in the US has been increasing drastically. Moreover, late preterm infants hold the responsibility for most of the increase. It is hypothesized that this increase is caused by the rise in multifetal pregnancies and use of reproductive technologies [10]. Based on our records in the Neonatal Intensive Care Department (NICD) in King Abdulaziz Medical City in 2018, there was an average of 9,099 deliveries per year, with an average of 1,094 preterm deliveries and an average of 792 late preterm deliveries. So, the late preterm infants represent about 9.5% of all the infants and 79% of the preterm infants admitted to our NICD. While previous studies worldwide have identified risk factors associated with higher readmission rates of late preterm infants, only a few studies have examined these risk factors in Saudi Arabia. However, the sample sizes of these studies were small, and they were limited by a lack of data on several variables. Therefore, this was the first study conducted at the National Guard Health Affairs to look for the rate of readmission in late preterm infants and associated risk factors.

## Materials and methods

Study design and area and settings

This was a retrospective cross-sectional study that was conducted at the Neonatal Intensive Care Department (NICD) at King Abdulaziz Medical City, Riyadh (KAMC-R). Ministry of National Guard Health Affairs in Saudi Arabia is a tertiary care department with a 40-bed Neonatal Intensive Care Unit (NICU) level IIIB, a 40-bed Intermediate Care Nursery (ICN) level II, and a 30-bed Special Care Baby Units (SCBU) level I. There are approximately 9,000 deliveries annually. The NICD is covered by 10 consultant neonatologists assisted by assistant consultants, staff physicians, and residents. Based on our records in the Neonatal Intensive Care Department (NICD) in KAMC in 2018, there is an average of 9,099 deliveries per year, with an average of 1,094 preterm deliveries and an average of 792 late preterm deliveries. This study focused on 792 late preterm infants of different nationalities. Late preterm infants born between 34 0/7 and 36 6/7 weeks of pregnancy in 2018 who were readmitted and stayed overnight in either the inpatient department or emergency room within the first month of discharge were the nominator. All the late preterm infants who are born between 34 0/7 and 36 6/7 weeks of pregnancy during the study period were the denominator. We calculated the required sample size using Raosoft software, assuming a margin of error of 5% and a confidence level of 95%. Based on a population of 792, the software determined that a sample size of 247 would be sufficient. This sample was selected using a non-probability consecutive sampling technique, given the retrospective nature of the study. We included the first 247 cases.

Data analysis and collection methods

The data were collected by revising the patients’ records (chart review) by the students and co-collector through the BESTCare system in National Guard Health Affairs to extract the dependent variable which is the readmission status along with its associated independent risk factors such as jaundice, feeding difficulties, and sepsis evaluation. As an example, we will consider any infant who was admitted and stayed overnight in either the inpatient department or emergency room as readmitted. Data were recorded using Excel (Microsoft, USA) and presented in the form of tables and graphs. R Studio software (version 2022.02.3, The R Foundation for Statistical Computing, Boston, USA) was used for data analysis. Categorical data such as readmission status, jaundice, feeding difficulties, and sepsis evaluation were presented as frequencies and percentages, while numerical data such as age and length of stay were presented as mean and standard deviation. The normality of data distribution was checked using the Kolmogorov-Smirnov test. Continuous normally distributed variables were presented as mean (± SD) and compared using an independent t-test. Continuous non-normally distributed variables were presented as median (25th percentile, 75th percentile) and compared using the Wilcoxon test. Categorical variables were presented as frequencies (%) and compared using Pearson’s Chi-squared test or Fisher’s exact test. Univariate logistic regression was done for all variables, and the odds ratio (OR, 95% confidence intervals) was reported.

Multivariate logistic regression analysis was used to correlate various factors with the risk of readmission. Factors reaching or approaching statistical significance (p ≤ 0.1) in the univariate analysis were included in the multivariate model. For all analyses, p < 0.05 was considered statistically significant.

## Results

**Figure 1:**
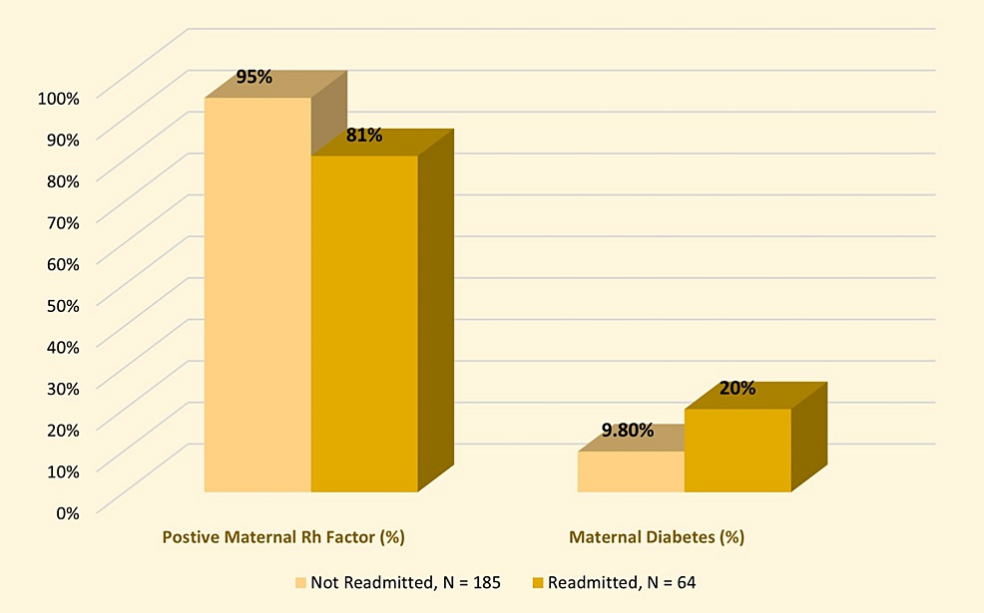
Clustered bar chart demonstrating significant maternal risk factors for readmission.

A total of 249 late preterm infants were included in the study. Of them, 64 infants (25.7%) suffered from at least a subsequent admission and stayed overnight in either the inpatient department or pediatric emergency room. Table 1 shows unadjusted maternal characteristics of readmitted versus not readmitted infants. A positive maternal Rh factor was a protective factor against readmission (95% vs. 81%; unadjusted odds ratio (OR) = 0.25 (0.1, 0.61); p = 0.002). Maternal diabetes was a significant risk factor for readmission (20% vs. 9.8%; unadjusted OR = 2.35 (1.06, 5.1); p = 0.032). Significant maternal risk factors are demonstrated in Figure 1. Both groups were comparable regarding maternal age, nationality, parity, obesity, hypertension, presence of other systemic illnesses, Coombs test positivity, blood group, short interpregnancy interval (<2 years), and mode of delivery.

**Table 1:** Unadjusted maternal characteristics of non-readmitted and readmitted infants. ^1^ Mean (±SD); n (%); Median (interquartile range (IQR)). ^2 ^OR: odds ratio, CI: confidence interval.

Characteristic	Not readmitted, N = 185^1^	Readmitted, N = 64^1^	Overall, N = 249^1^	N	OR^2^	95% CI^2^	p-value
Maternal age (years)	32 (±6)	31 (±7)	32 (±6)	248	0.97	0.93, 1.02	0.3
Parity				247			
Nullipara	53 (29%)	19 (30%)	72 (29%)		-	-	
Multipara	130 (71%)	45 (70%)	175 (71%)		0.97	0.52, 1.83	>0.9
Maternal Rh factor				247			
Negative	10 (5.5%)	12 (19%)	22 (8.9%)		-	-	
Positive	173 (95%)	52 (81%)	225 (91%)		0.25	0.10, 0.61	0.002
Diabetes	18 (9.8%)	13 (20%)	31 (12%)	248			
No					-	-	
Yes					2.35	1.06, 5.10	0.032
Short interpregnancy interval (<2 years)	49 (29%)	14 (24%)	63 (28%)	225			
No					-	-	
Yes					0.77	0.38, 1.50	0.4
Mode of delivery				247			
Vaginal	85 (46%)	34 (53%)	119 (48%)		-	-	
Cesarean	98 (54%)	30 (47%)	128 (52%)		0.77	0.43, 1.35	0.4

**Figure 2:**
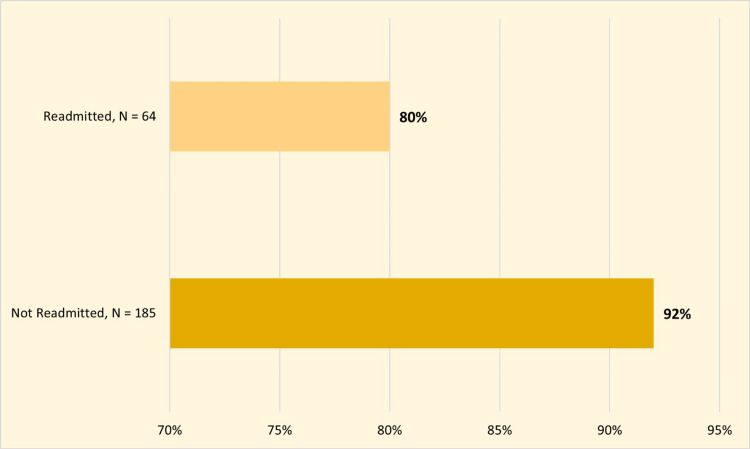
Clustered bar chart demonstrating difference in breastfeeding among both study groups.

Unadjusted demographic and birth characteristics of non-readmitted and readmitted infants are shown in Table 2. Breastfeeding was a protective factor against readmission (92% vs. 80%; unadjusted OR = 0.36 (0.14, 0.92); p = 0.03; Figure 2). There was no statistically significant difference between both groups regarding infantile gender, nationality, weight, length, gestational age, age at NICU discharge, blood group, Rh factor, Coombs test, Apgar score at 1 minute, and Apgar score at 5 minutes.

**Table 2:** Unadjusted demographic and birth characteristics of non-readmitted and readmitted infants. ^1 ^Mean (±SD); n (%); median (interquartile range (IQR)). ^2 ^OR: odds ratio, CI: confidence interval.

Characteristic	Not readmitted, N = 185^1^	Readmitted, N = 64^1^	Overall, N = 249^1^	N	OR^2^	95% CI^2^	p-value
Gender				249			
Male	89 (48%)	23 (36%)	112 (45%)		-	-	
Female	96 (52%)	41 (64%)	137 (55%)		1.65	0.93, 3.00	0.093
Weight (kg)	2.44 (±0.45)	2.51 (±0.52)	2.46 (±0.47)	247	1.41	0.77, 2.62	0.3
Length (cm)	47.0 (44.0, 49.0)	46.0 (42.8, 49.0)	47.0 (43.0, 49.0)	246	0.99	0.94, 1.05	0.7
Breastfeeding	126 (92%)	41 (80%)	167 (89%)	188			
No					-	-	
Yes					0.36	0.14, 0.92	0.030
Gestational age (weeks)	36 (35:36)	35 (35:36)	36 (35:36)	248	0.79	0.54, 1.16	0.2
Age at NICU discharge (days)	4 (3:9)	6 (3:11)	5 (3:10)	236	1.01	0.98, 1.04	0.4
Infant Rh factor				248			
Negative	20 (11%)	9 (14%)	29 (12%)		-	-	
Positive	164 (89%)	55 (86%)	219 (88%)		0.75	0.33, 1.81	0.5
Apgar score at 1 minute	9 (8:9)	9 (8:9)	9 (8:9)	244	0.96	0.79, 1.20	0.7
Apgar score at 5 minutes	9 (9:9)	9 (9:9)	9 (9:9)	244	1.16	0.72, 2.50	0.6

**Figure 3:**
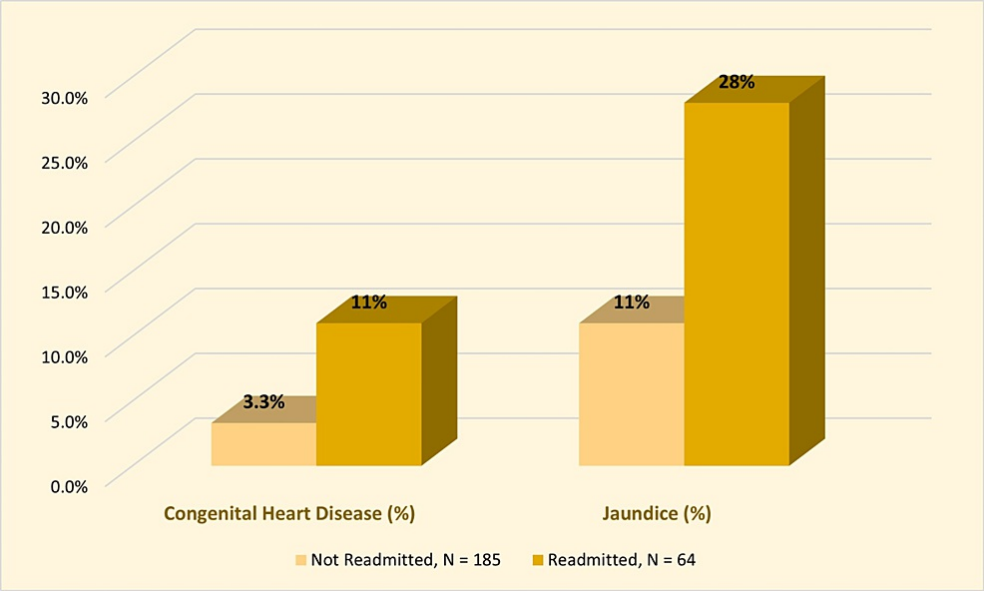
Clustered bar chart demonstrating fetal conditions associated with a higher risk of readmission.

Table 3 shows unadjusted clinical characteristics of non-readmitted and readmitted infants. Congenital heart disease (CHD) was a significant risk factor for readmission (11% vs. 3.3%; OR = 3.64 (1.17, 11.7); p = 0.025). Jaundice was also a significant risk factor for readmission (28% vs. 11%; OR = 3.21 (1.5, 6.59); p = 0.001; Figure 3). Both groups were comparable regarding the length of stay, neonatal respiratory distress syndrome (NRDS), bronchiolitis, apnea, URTI, other respiratory problems, anemia, cardiac problems other than CHD, gastroesophageal reflux disease (GERD), enteritis, other GIT problems, endocrine problems, Down syndrome, G6PD deficiency, other congenital problems, UTI, CNS problems, cyanosis, feeding problems, dehydration, and other general problems.

**Table 3:** Unadjusted clinical characteristics of non-readmitted and readmitted infants. ^1^ Mean (±SD); n (%); median (interquartile range (IQR)). ^2^ OR: odds ratio, CI: confidence interval, NA: not applicable.

Characteristic	Not readmitted, N = 185^1^	Readmitted, N = 64^1^	Overall, N = 249^1^	N	OR^2^	95% CI^2^	p-value
Length of stay (days)	4 (2:10)	6 (4:8)	5 (2:9)	101	0.97	0.88, 1.05	0.5
Neonatal respiratory distress syndrome (NRDS)	37 (20%)	11 (18%)	48 (20%)	244			
No					-	-	
Yes					0.85	0.39, 1.73	0.7
Apnea	1 (0.5%)	1 (1.6%)	2 (0.8%)	249			
No					-	-	
Yes					2.92	0.11, 74.6	0.5
Upper respiratory tract infection (URTI)	4 (2.2%)	1 (1.6%)	5 (2.0%)	248			
No					-	-	
Yes					0.73	0.04, 5.05	0.8
Congenital heart disease (CHD)	6 (3.3%)	7 (11%)	13 (5.2%)	248			
No					-	-	
Yes					3.64	1.17, 11.7	0.025
Enteritis	5 (2.7%)	1 (1.6%)	6 (2.4%)	248			
No					-	-	
Yes					0.57	0.03, 3.61	0.6
Other gastrointestinal problems	7 (3.8%)	6 (9.4%)	13 (5.2%)	248			
Unknown	1	0	1				
No					-	-	
Yes					2.62	0.81, 8.18	0.10
Endocrine problems	1 (0.5%)	1 (1.6%)	2 (0.8%)	248			
No					-	-	
Yes					2.90	0.11, 74.2	0.5
Down syndrome	2 (1.1%)	1 (1.6%)	3 (1.2%)	248			
No					-	-	
Yes					1.44	0.07, 15.3	0.8
G6PD deficiency	1 (0.5%)	1 (1.6%)	2 (0.8%)	248			
No					-	-	
Yes					2.90	0.11, 74.2	0.5
Urinary tract infection (UTI)	3 (1.6%)	0 (0%)	3 (1.2%)	248			
No					-	-	
Yes					0.00		>0.9
Sepsis	23 (12%)	8 (12%)	31 (12%)	248			
No					-	-	
Yes					1.00	0.40, 2.28	>0.9
CNS problems	4 (2.2%)	2 (3.1%)	6 (2.4%)	248			
No					-	-	
Yes					1.45	0.20, 7.63	0.7
Cyanosis	1 (0.5%)	2 (3.1%)	3 (1.2%)	248			
No					-	-	
Yes					5.90	0.56, 128	0.2
Jaundice	20 (11%)	18 (28%)	38 (15%)	248			
No					-	-	
Yes					3.21	1.56, 6.59	0.001
Feeding problems	0 (0%)	1 (1.6%)	1 (0.4%)	248			
No					-	-	
Yes					6,186,430	0.00, NA	>0.9

Among readmitted infants (n = 64), 51 infants were readmitted to the emergency room (79.69%), eight infants were readmitted to the pediatric ward (12.5%), and five infants were readmitted to both (7.8%). As shown in Table 4, the most common cause for ER admission was GIT problems (27%) followed by URTI (18%), jaundice (14%), airway problems (12%), pyrexia (12%), orthopedic problems (3.9%), and CHD (3.9%). Other known causes for ER admission constituted 9.8%. All those infants were discharged after improvement without pediatric ward admission.

**Table 4:** Readmission characteristics. ^1 ^n (%). ^2^Fisher's exact test. GIT: gastrointestinal, URTI: upper respiratory tract infection, CHD: congenital heart disease, CDH: congenital diaphragmatic hernia.

Characteristic	ER, N = 51^1^	Ward, N = 8^1^	Both, N = 5^1^	Overall, N = 64^1^	p-value^2^
Cause of readmission					0.003
GIT problem	14 (27%)	0 (0%)	0 (0%)	14 (22%)	
Jaundice	7 (14%)	5 (62%)	0 (0%)	12 (19%)	
URTI	9 (18%)	0 (0%)	3 (60%)	12 (19%)	
Airway problem	6 (12%)	1 (12%)	2 (40%)	9 (14%)	
Pyrexia	6 (12%)	0 (0%)	0 (0%)	6 (9.4%)	
Others	5 (9.8%)	0 (0%)	0 (0%)	5 (7.8%)	
CHD	2 (3.9%)	0 (0%)	0 (0%)	2 (3.1%)	
Orthopedic problem	2 (3.9%)	0 (0%)	0 (0%)	2 (3.1%)	
CDH	0 (0%)	1 (12%)	0 (0%)	1 (1.6%)	
Regurgitation	0 (0%)	1 (12%)	0 (0%)	1 (1.6%)	

Jaundice was the most common cause of direct ward readmission (n = 5; 62%). The other three infants who were directly readmitted to the ward without ER readmission had airway problems, congenital diaphragmatic hernia (CDH), and regurgitation. Three infants with URTI and two infants with airway problems were readmitted to ER and then transferred to the ward. Of all readmissions, GIT problems were the most common cause (22%), followed by jaundice (19%), URTI (19%), and airway problems (9.4%).

To adjust for all maternal, demographic, birth, and clinical characteristics, a multivariate logistic regression model was built (Table 5). This model aimed to investigate potential risk factors for late preterm infant readmission. Any variable with a significant p-value was included in this model. Although this model was found to be highly statistically significant (p < 0.001) and could account for 45% of the variation in infant readmission, none of the included factors alone contributed significantly to the risk of readmission. In other words, the interactions of those factors rather than an individual factor contributed to the risk of readmission.

**Table 5:** Multivariate regression model for all readmissions. ^1^aOR: adjusted odds ratio, CI: confidence interval.

Characteristic	aOR^1^	95% CI^1^	p-value
Maternal Rh factor			
Negative	-	-	
Positive	0.45	0.15, 1.40	0.2
Diabetes			
No	-	-	
Yes	1.85	0.68, 4.88	0.2
Infant age (days)	1.00	1.00, 1.01	0.087
Breastfeeding			
No	-	-	
Yes	0.58	0.19, 1.78	0.3
Congenital heart disease (CHD)			
No	-	-	
Yes	1.41	0.28, 6.61	0.7
Jaundice			
No	-	-	
Yes	2.10	0.90, 4.80	0.080

## Discussion

Late preterm infants encompass the majority of preterm infants, and they are predisposed to many risk factors that can increase their readmission rate. In our research, we have found that GIT issues and URTI are the most common causes that lead to pediatric ER admission, while jaundice was the main cause of ward readmission. Compared to term infants, late preterm infants had a higher incidence of hyperbilirubinemia. Several documented studies that have been published indicate that there is a correlation between the increase in jaundice with inadequate breastfeeding [11]. A previous study has demonstrated the length of stay to be proportionally related to readmission as each additional day in the length of stay was associated with a reduced risk of readmission. Yet, the relation between the two was insignificant in this study [5]. Moreover, maternal age, parity, and mode of delivery were proven to increase the infants' risk of readmission. On the other hand, we have not detected any correlation in our data [1]. One possible explanation is that our study had different demographic and clinical characteristics compared to the previous study. While we did not find a significant correlation between these factors and infants' risk of readmission, we believe that further research is needed to fully understand their impact on this outcome.

Many other risk factors besides GIT problems accounted for readmission to the neonatal intensive care unit within the first month of life. These factors include jaundice, upper respiratory tract infections, airway problems, pyrexia, and congenital heart diseases. However, there were obvious disparities in their frequency and intensity in the affected neonates, and the variations could be attributed to individualized factors in the neonates. In contrast to our findings, a previously published local study failed to report similar causes for the readmission [1]. A population-based cohort study by Tomashek et al. [12] evaluated the hospital readmission rate of late preterm infants in the neonatal period.

In this study, the most common cause for pediatric ER admission was GIT problems, followed by URTI, jaundice, airway problems, pyrexia, orthopedic problems, and CHD. On the other hand, jaundice was the most common cause for direct ward admission (without a referral from the pediatric ER). One infant was admitted for an airway problem and another one for regurgitation, which is comparable to a few articles that mentioned that the most common risk factors for readmission within the first month were jaundice, acute bronchiolitis, viral infections, congenital heart diseases, and respiratory infections [13-14]. In contrast, other studies demonstrated that feeding problems were the primary risk factor leading to readmission within the first month [15]. The limitation of this retrospective cross-sectional study is the potential for selection bias, as the study population is based on patients' medical records rather than a randomized sample. Additionally, the study relies on the accuracy and completeness of the recorded data, which may be subject to errors or missing information.

## Conclusions

This study aims to investigate the common causes of pediatric emergency department (ER) and ward admissions among late preterm infants, defined as infants born between 34+0 and 37+6 weeks. It is known that late preterm infants are at higher risk of developing multiple diseases due to both fetal and maternal factors. In this study, the most frequent cause of pediatric ER admission was gastrointestinal (GIT) problems, followed by upper respiratory tract infections (URTIs). Conversely, jaundice was the leading cause of ward admission, followed by congenital diaphragmatic hernia (CDH), airway problems, and regurgitation. The study also highlights the need for further research to fully understand the long-term outcomes for this vulnerable population.
